# Sex-trafficking, Violence, Negotiating Skill, and HIV Infection in Brothel-based Sex Workers of Eastern India, Adjoining Nepal, Bhutan, and Bangladesh

**Published:** 2008-06

**Authors:** Kamalesh Sarkar, Baishali Bal, Rita Mukherjee, Sekhar Chakraborty, Suman Saha, Arundhuti Ghosh, Scott Parsons

**Affiliations:** ^1^National Institute of Cholera & Enteric Diseases, P-33 CIT Road, Scheme XM, Beliaghata, Kolkata 700 010, India; ^2^Daywalka Foundation, 9/B Hazi Mahasin Road, Kolkata 700 026, India; ^3^Daywalka Foundation, Hatfield School of Government, Portland States University, Portland, OR, USA

**Keywords:** Acquired immunodeficiency syndrome, Community-based studies, Cross-sectional studies, HIV infections, Negotiating skills, Prostitutions, Sex-trafficking, Sex worker, Sexually transmitted diseases, Violence, Bangladesh, Bhutan, India, Nepal

## Abstract

A community-based cross-sectional study was conducted among brothel-based sex workers of West Bengal, eastern India, to understand sex-trafficking, violence, negotiating skills, and HIV infection in them. In total, 580 sex workers from brothels of four districts participated in the study. A pretested questionnaire was introduced to study their sociodemography, sex-trafficking, violence, and negotiating skills. Blood sample of 4–5 mL was collected from each sex worker using an unlinked anonymous method to study their HIV status. Data were edited and entered into a computer using the Epi Info software (6.04d version). Both univariate and multivariate analyses were done to find out any association between HIV and relevant risk factors. Results of the study revealed that a sizeable number of the participants were from Nepal (9%) and Bangladesh (7%). The seroprevalence of HIV was strikingly higher among Nepalese (43%) than among Bangladeshis (7%) and Indians (9%). Almost one in every four sex workers (24%) had joined the profession by being trafficked. Violence at the beginning of this profession was more among the trafficked victims, including those sold by their family members (57%) compared to those who joined the profession voluntarily (15%). The overall condom negotiation rate with most recent two clients was 38%. By multivariate analysis, HIV was significantly associated with sexual violence (odds ratio=2.3; 95% confidence interval 1.2–4.5). The study has documented that the trafficked victims faced violence, including sexual violence, to a greater magnitude, and sexual violence was associated with acquiring HIV in them. There is a need for an in-depth study to understand the problem of trafficking and its consequences.

## INTRODUCTION

West Bengal, one of the eastern states of India, with a population of more than 80 million (1), has not recognized HIV much as a problem till recently and was considered to be one of the low-prevalent states in the context of HIV/AIDS in India (2,3). The state has three international boundaries with neighbouring countries, such as Nepal, Bhutan, and Bangladesh, through which continuous migration of population, especially young females, takes place. Migration can place women in situations where they experience stress and anxiety due to the loss of their traditional social entourage and environment. Rape and sex work among migrant women become the key factors in the transmission of HIV/AIDS and sexually transmitted diseases (4).

Nepal is known to be a country from which HIV has spread to injecting drug users (IDUs) in the adjoining areas of West Bengal, eastern India, and across the Indo-Nepal border (5). The state is also connected to the high HIV-prevalent northeastern states, such as Nagaland and Manipur (6), through its northern bordering town—Siliguri. On the other hand, Kolkata, the state capital, is one of the four major metropolitan cities of the country with a total population (including floating population) of more than 10 million (1). An estimated 50,000 sex workers operate in West Bengal, and about half of them work in the capital city of Kolkata (7).

Earlier research by the National Institute of Cholera & Enteric Diseases (NICED) conclusively established that young female sex workers aged less than 20 years are more likely to be HIV-positive than the sex worker population at large (8). On the other hand, young girls from low socioeconomic families are vulnerable to trafficking, primarily for sex work, particularly in countries, such as India, Nepal, and Bangladesh, where a sizeable number of people do not have much income to support them. They are also susceptible to frequent violence and injuries caused by the traffickers and their network members. This leads to more unsafe or unprotected sexual practices with their clients. All the above factors combined with a biological condition, like a larger area of cervical ectopy, are associated with a higher rate of HIV infection among young sex workers (9-11). This could be further fuelled by sexual violence and/or genital injury that are often associated with them, who are trafficked and forced to join the sex trade.

Hence, a study was conducted to understand sex-trafficking, violence, negotiating skills, and HIV infection in brothel-based sex workers of West Bengal.

## MATERIALS AND METHODS

### Study method

It was a community-based cross-sectional study. Sex workers of four district brothels in West Bengal were approached through local non-governmental organizations (NGOs) and community-based organizations working with them. Initially, all sex workers were asked to participate voluntarily after explaining the purpose of the study to them. In total, 580 sex workers who responded voluntarily from the selected brothels were included in the study. Verbal consent was obtained from them. A pretested questionnaire was introduced to the subjects to study their sociodemographic variables, trafficking, violence, negotiating skill and other risk behaviours by three experienced social workers under the supervision of a sociologist. Sex-trafficking was asked by putting a question like “How did you join this profession?” Response options included: (a) voluntarily, (b) forced by family member(s), and (c) misguided/forced by other less-known/unknown/distant relative(s). Violence was questioned by “Did you face any torture (physical/mental/sexual), including rape, during initial few weeks of joining sex work?”. Negotiating skill was asked by putting question like “Could you use condom with your most recent two clients—yes/no; if yes, please specify separately whether it was self-use by the client himself or used only after negotiation.”

### Sample collection and processing

Interview was followed by collection of 4–5-mL blood samples using an unlinked anonymous method. Blood samples were collected in a vacutainer and transported to the virology laboratory of NICED, Kolkata, one of the national HIV referral laboratories of eastern India. Testing of HIV was done by enzyme-linked immunosorbent assay (ELISA), followed by another rapid test (tri-dot test) as per the national guidelines of HIV testing. ELISA tests were done by the Vironostika HIV Uni-Form II Ag/Ab Microelisa System. The test was performed according to the instructions of the manufacturer (Biomerieux, Boseind-15, 5281RM Boxtel, The Netherlands), and samples reactive for tests were detected as positive for HIV. The ELISA-reactive samples were then retested with another immuno-blot method, known as ‘HIV-TRI DOT’, as per the instructions of its manufacturer (J. Mitra & Co. Ltd., New Delhi, India).

### Data management

Once data arrived from the field, these were entered into a computer using the Epi Info software (6.04d version) after editing these carefully. Once all the entries, including HIV test reports of 580 samples, were completed, univariate and multivariate analyses were done considering HIV as an independent variable and factors, such as age, trafficking, violence, negotiating skills, etc., as dependent variables. The variables that showed a statistical association on univariate analysis were selected for multivariate analysis.

**Fig. 1:**
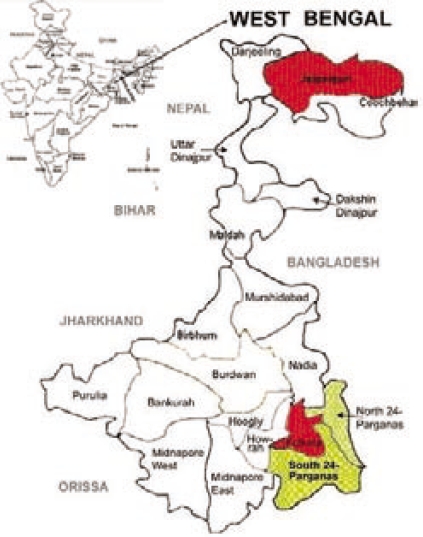
Studied districts of West Bengal with their locations in India (inset)

The studied districts of West Bengal were Kolkata, 24 Parganas (North), Jalpaiguri, and 24 Parganas (South) (Fig. 1). The duration of the study was six months from May to October 2006.

### Ethics

Ethical clearance was obtained from the Institutional Review Board of NICED and Daywalka Foundation, India, prior to initiating the study.

## RESULTS

**Fig. 2:**
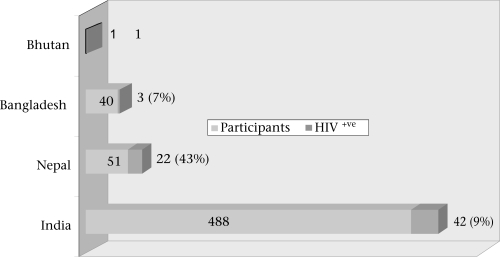
Country-wise distribution of studied sex workers (n=580) with HIV status

Of the 580 sex workers from brothels of four different districts of West Bengal, who participated in the study, 488 (84%) were from India, 51 (9%) from Nepal, 40 (7%) from Bangladesh, and only one from Bhutan. Overall, the rate of HIV seroprevalence was 12% (n=68) among the participants. Figure 2 shows the distribution of the study participants with their country of origin and corresponding seroprevalence of HIV among them.

The figure shows that sex workers from Nepal had the HIV seroprevalence rate of 43%. Sex workers of Indian origin had an HIV seroprevalence rate of 9%, and Bangladesh had an HIV seroprevalence rate of 7%. The lone sex worker from Bhutan was infected with HIV.

**Fig. 3:**
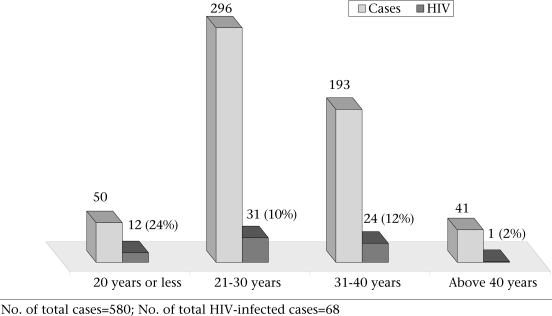
Age distribution of studied sex workers (n=580) with corresponding HIV status

The commonest age-group was 21–30 years (51%; n=296) with the mean age of 29.8 years. The studied participants aged 20 years or less were 9% (n=50). The seroprevalence of HIV was the highest (24%) among sex workers aged 20 year or less. The same was 10% in the age-group of 21–30 years and 12% in the age-group of 31–40 years. The seroprevalence of HIV was the lowest (2%) among participants aged over 40 years (Fig. 3). The mean number of clients entertained daily by sex workers aged 20 years or less was the highest (3.66 clients/day) compared to that of the 21–35-year age-group (2.67 clients/day) and above 35-year age-group (1.65 clients/day).

Forty-six percent of the participants started sex work at the age of 16–20 years. This was followed by 25% of them initiating at the age of 21–25 years. About 10% initiated their sex work at the age of 15 years or below. The overall mean age of initiation of sex work was 21.2 years.

Thirty-two percent of the subjects had sex work for a duration of 1–5 year(s); about 28% had sex work for a duration of >5–10 years; about 11% had it for a duration of one year or less; and 7% had it for a duration of more than 20 years. The mean duration of sex work among all the study participants was 8.6 years. The majority (58%) of the sex workers entertained 1–2 client(s) daily, followed by 3–4 clients (34%). Only 1% entertained seven or more clients, whereas the remaining 7% entertained 5–6 clients daily.

The majority (68%; n=397) of the sex workers joined the profession voluntarily and primarily due to poverty. About 24% found themselves cheated and were forced by somebody to join the profession against their will. These were the girls who were trafficked and brought into sex work. The remaining 8% were forced to join the profession directly or indirectly by their spouses or family members. It has also been observed that 46% of 50 sex workers aged 20 years or less had the experience of trafficking compared to 30% (n=160) of older sex workers aged over 20 years having a similar experience.

Results of the study showed that violence was faced more by the trafficked sex workers, including those sold by their families (57%) compared to those who joined voluntarily (15%). The seroprevalence of HIV was 13% (n=24) among the trafficked sex workers, including those sold by their families compared to those who joined the profession voluntarily (10%; n=40).

Table 1 shows that 63% (n=105) of the sex workers who faced violence (n=166) in their early professional sex work were victims of trafficking. On the other hand, 37% (n=61) had the history of violence despite joining the profession voluntarily. This difference was statistically significant (odds ratio [OR]=7.4; confidence interval [CI] 95% 4.8–11.3).

**Table 1:** Violence and trafficked victims

Violence	No. of trafficked victims, including those sold by family members	No. who joined voluntarily	Odds ratio (95% confidence interval)
Suffered from violence	105	61	7.4
Did not suffer from violence	78	336	(4.8–11.3)

Of the trafficked victims, 66% were assured with jobs, and 27% were assured of better quality of life. Promise of marriage was assured to about 4% of the study participants, whereas 3% of those who did not join the profession voluntarily (n=183) had a single recruiter (88%; n=161), and 12% had two recruiters (n=22).

About 29% (n=166) of the sex workers faced violence during the early phase of their profession. The seroprevalence of HIV was 20% (n=33) among those who faced violence during the initial phase. The same was 8% among those who did not face violence during the similar period.

Of the 166 sex workers who faced violence, 15% faced physical violence, 55% had sexual violence, and 30% had both. Both clients and brothel owners or their representatives committed physical and sexual violence in most cases. About 16% (n=92) of the participants were forced to do sex against their will initially as part of sexual violence. Recruiters, brothel owners, or their representatives were involved in most cases. Pimps and customers were also associated with 13% and 6% of the cases respectively. The seroprevalence of HIV was 23% (n=32) among sex workers who faced sexual violence initially compared to 8% (n=36) among those who did not.

Ideal time of negotiation for condom-use appears to be better after taking the client on bed, when client begins to be intimated with sex workers. Acceptance of condom-use appears to be better at this phase for unwilling clients. However, in this study, only 38% of the participants had practised it at that phase, i.e. after taking them to bed. The majority (55%) of the sex workers did it during price-fixing, which appeared to be less effective compared to the former approach. The seroprevalence of HIV was less (9%; n=20) among sex workers who negotiated after taking them to bed compared to those who did not (13%; n=44).

**Fig. 4:**
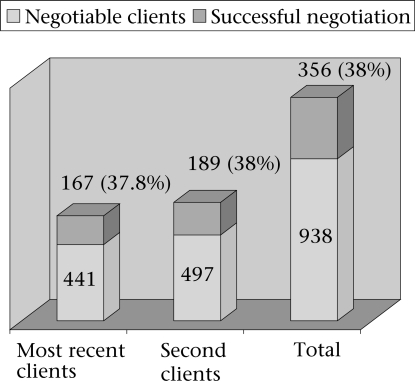
Negotiated condom-use rates with most recent two clients (n=566)

Most (86%; n=499) sex workers asked their clients to use condom as a routine practice. Regarding the assessment of negotiating skill, sex workers were asked about condom-use with their most recent two clients. If the response was ‘yes' with any one of them, the respondent was asked again whether it was with or without negotiation as some clients used condoms voluntarily and did not require any negotiation. In total, 566 participants responded to this question. In total, 292 and 258 sexual acts took place with condoms with the first client (most recent) and the second client respectively. Of the 292 sexual acts using condoms with the first client, 125 clients used condoms voluntarily and did not require any negotiation. So, actual scope of negotiation for condom-use with the first client was (566–125) 441. Of the 441 clients who did not have any intention to use condoms, the sex workers negotiated successfully with 167 clients. This gives rise to a rate of 38% (167 of 441) as their successful condom negotiation rate. In this way, the rate was 38% with their second clients. So, the overall successful condom negotiation rate was 38% (rounded up) among the studied participants (Fig. 4).

Most (97%; n=563) sex workers did not have any formal training on negotiating skill; only 4% participated in some kind of training on negotiating skill organized by local NGOs.

In total, 321 (55%) of the 580 subjects had the history of consumption of alcohol. Of the 321 alcohol drinkers, 233 (73%) consumed it in varying quantity and frequency before entertaining their clients. Cost of alcohol was borne by the clients for most (74%; n=238) sex workers. Negotiation for condom-use seemed to be affected, at least to some extent, when sex was offered with prior alcohol intake.

Univariate analysis was done considering HIV as an independent variable and younger age of less than 20 years, trafficking, violence, sexual violence, forced to perform sex against will, timing of negotiation, etc. as dependent variables. HIV was associated with younger age (20 years or less) (OR=2.6; CI 1.2–5.6), trafficking (OR=1.9; CI 1.1–3.3), violence faced during early phase (OR=2.6), sexual violence (OR=3.2; CI 1.8–5.7), and forced sex against will (OR=3.0; CI 1.6–5.5). HIV was not, however, associated with negotiation for condom-use during entry or price-fixing. In multivariate analysis, sexual violence was found to be associated with HIV infection (OR=2.3; 95% CI 1.2–4.5) (Table 2).

**Table 2:** Univariate and multivariate analyses showing factors associated with HIV

Variable	HIV^+ve^ cases	HIV^−ve^ cases	Odds ratio (95% CI) (Univariate analysis)	Odds ratio (95% CI) (Multivariate analysis)
Younger age of 20 years or less (n=50; 8.6%)	12	38	2.6 (1.2–5.6)	1.6 (0.7–3.5)
Joined sex work being trafficked	24	115	1.90 (1.1–3.3)	1.05 (0.5–1.9)
Violence faced during early phase of profession	33	133	2.6 (1.5–4.6)	1.68 (0.7–2.1)
Sexual violence faced during early phase of profession	32	109	3.2 (1.8–5.7)	2.36 (1.2–4.5)
Forced to perform sex against will during initial phase of joining	22	70	3.0 (1.6–5.5)	1.59 (0.7–3.2)
Negotiation for condom-use at entry or during price fixing	44	302	1.4 (0.8–2.2)	0.82 (0.4–1.4)

## DISCUSSION

Because of geographical proximity, a large number of girls from the neighbouring countries, such as Nepal and Bangladesh, settled in brothels of different districts of West Bengal for sex work. In this study, of the 580 participants, 51 were from Nepal, 40 were from Bangladesh, and only one was from Bhutan. This indicates cross-border migration of girls for sex work, and a sizeable number could be through trafficking as similar observations were reported in other studies (12-15). The seroprevalence of HIV was higher (43%; n=22) among Nepalese compared to Bangladeshis (7%; n=3) and Indians (9%; n=42). Only one participant from Bhutan was found to be positive with HIV. This higher rate of HIV among Nepalese sex workers indicate that they are more vulnerable, probably because of their higher number joining the profession through sex-trafficking (16) and entering the profession at a younger age as revealed by another study that found that the median age of trafficking of Nepalese girls was 17 years (17). Younger sex workers were more vulnerable compared to older age-groups. This could be due to a larger area of cervical ectopy along with behavioural factors, such as less empowerment and poor negotiating skill of younger sex workers. It has also been observed in the study that sex workers aged 20 years or less had entertained a higher number of clients daily (mean number=3.66 clients/day) compared to older age-groups. More sexual activity in younger girls leads to repeated trauma to immature genital tract that facilitates the transmission of HIV. The present study has shown a higher prevalence of HIV among younger sex workers aged less than 20 years compared to older age-groups (Fig. 3). The age of 20 years has been taken as a cut-off point in the present study due to the fact that this was a brothel-based study, and sex workers aged less than 18 years (legally considered as minor) are not allowed to work in brothels of West Bengal at present. The problem of minor girls aged less than 18 years could better be studied at rescue homes where trafficked victims, including minor girls, are kept for a varying length of time after rescuing them when legal procedures are made before sending them back to their homes.

Human trafficking is not only a problem of developing countries but also of developed countries. An estimated 50,000 women and children are annually trafficked into the United States, resulting in complex health and social consequences and significant risk for violence (18). Among Asian countries, human trafficking is really a major concern in India, Bangladesh, and Nepal, primarily because of illiteracy and economic dependency. In this study, about one-fourth (24%; n=139) reported that they joined the profession after being trafficked. The majority (86%; n=120) of the victims were trafficked as minors and by individuals previously known to them. The traffickers most commonly lured victims by offering promises of economic opportunity or kidnapped individuals using drugs or force. Victims were most often trafficked from public settings, e.g. markets and railway stations, and via public transportation (19). In this study, the seroprevalence of HIV was much higher among the trafficked sex workers (17%) compared to the non-trafficked ones (10%; n=441). This difference was statistically significant on univariate analysis (OR=1.9; CI 1.1–3.3) (Table 1) but not on multivariate analysis.

In this study, violence was observed in 29% (n=166) of the participants during their initial phase of joining sex work. Violence was observed to be more among the trafficked victims, including those sold by their family members compared to those who joined the profession voluntarily. This difference was statistically significant (Table 1). This indicates that the trafficked victims are more at risk of violence. The higher seroprevalence of HIV was observed among those who suffered from violence initially compared to those who did not. This difference was statistically significant in univariate analysis (OR=2.6; CI 1.5–4.6) (Table 2). The prevalence of sexual violence (faced initially) among the participants was 24% (n=141). Considering sexual violence faced initially, the seroprevalence of HIV was considerably higher among them (23%; n=32) compared to those who did not have (8%; n=36). This difference was statistically significant by both univariate (OR=3.2; CI 1.8–5.7) and multivariate analyses (OR=2.3; CI 1.2–4.5). This indicates that sex workers who faced sexual violence were 2.3 times more at risk of acquiring HIV compared to those who did not. Results of a study in Bangalore, India, reported a higher HIV seropositivity among women with domestic violence (20). Results of a study in the USA had observed that the rate of HIV positivity was very high among women who experienced more frequent and severe violence from their intimate partners (21). Results of several studies in Africa showed that high level of sexual violence was associated with more unsafe sexual practices and HIV (22-24). Traumatized women engaging in substance abuse and unsafe sex were also at high risk for contracting HIV/AIDS as observed in another study (25). Findings of all these studies support the findings of the present study that sex workers having sexual violence are significantly associated with HIV.

Acquiring negotiating skill for condom-use by clients appeared to be an important strategy for HIV intervention, particularly in the absence of a suitable female-controlled HIV intervention device. No standard and uniform method exists for evaluating negotiating skills of sex workers, and it depends on factors, such as skill, knowledge, and attitudes of sex workers with or without influence of alcohol. In this study, successful negotiation rate was observed in 38% of the participants with their most recent and second clients. This indicates that most (62%) sex workers were unable to negotiate with their unwilling clients. The reason could be primarily due to avoidance of loss of income leading to unprotected sex with their clients as awareness of HIV was high in sex workers of West Bengal (26). Inconsistent condom-use among sex workers has also been documented to be high in other parts of India, leading to higher vulnerability to HIV (27). Another important factor that could play a significant role on negotiation is prior consumption of alcohol either by clients or by sex workers or by both. In this study, 55% (n=321) of the study participants consumed alcohol. Of them, 73% (n=233) consumed it at clients' account before entertaining them. Influence of alcohol on negotiating skills needs to be studied further in-depth to find out some suitable behavioural intervention mechanisms targeted towards them.

It is evident that an in-depth study, including finding a suitable method to reach those at a greater risk—young and trafficked victims—is required. The present model of the study, although highlighted the trafficking and its consequences, may not be adequate as the event occurred retrospectively. A more appropriate and feasible method could have been applied in the study of trafficked victims as soon as possible following the detection of events. The said trafficked victims could be approached in the rescued homes where they are kept for a variable length of time after rescuing them from trafficking.

Convenient sampling, self-reported behaviour, non-availability of minor girls in brothels as study subjects, interviewing participants in brothels where privacy and suitable spaces were not available in some cases, apprehension of revealing unwanted information of brothel owner, loss of business hours and recall bias in some cases were important limitations of the study.

The study has primarily documented the factors, such as trafficking, violence, and negotiating skills of sex workers that play a role in acquiring HIV infection. Among the participants, a sizeable number was from Nepal and Bangladesh. The seropositivity rate of HIV was strikingly higher among Nepalese, which could be related to a high number of trafficking of girls from Nepal at their early ages. It is alarming that 24% of the brothel-based sex workers joined their work by being trafficked. The study has documented that trafficked victims were vulnerable to violence, and sexual violence was significantly associated with HIV. It is also interesting to note that negotiating skill was found to be 38% in sex workers of West Bengal. It appeared that sex-trafficking was an important factor for acquiring HIV/AIDS in sex workers. An in-depth study is required to understand the different dimensions of sex-trafficking further and for designing a suitable intervention strategy.
